# The Need for Pediatric Formulations to Treat Children with HIV

**DOI:** 10.1155/2016/1654938

**Published:** 2016-06-16

**Authors:** Adrienne F. Schlatter, Andrew R. Deathe, Rachel C. Vreeman

**Affiliations:** ^1^Department of Pediatrics, Indiana University School of Medicine, Indianapolis, IN 46202, USA; ^2^Academic Model Providing Access to Healthcare (AMPATH), Eldoret 30100, Kenya

## Abstract

Over 3.2 million children worldwide are infected with HIV, but only 24% of these children receive antiretroviral therapy (ART). ART adherence among children is a crucial part of managing human-immunodeficiency virus (HIV) infection and extending the life and health of infected children. Important causes of poor adherence are formulation- and regimen-specific properties, including poor palatability, large pill burden, short dosing intervals, and the complex storage and transportation of drugs. This review aims to summarize the various regimen- and formulation-based barriers to ART adherence among children to support the need for new and innovative pediatric formulations for antiretroviral therapy (ART). Detailing the arguments both for and against investing in the development of pediatric HIV medications, as well as highlighting recent advances in pediatric ART formulation research, provides a synopsis of the current data related to pediatric ART formulations and adherence.

## 1. Background

Over 3.2 million children worldwide are infected with HIV, but only 24% of these children are on the antiretroviral therapy (ART) they need [1]. Ninety percent of children living with HIV reside in sub-Saharan Africa, and HIV/AIDS is the most common cause of death for adolescents in Africa and the second most common cause of death for adolescents worldwide [2]. At the 2015 United Nations' Summit, the World Health Organization (WHO) lifted age and medical restrictions for ART initiation [3]. The WHO now recommends that all individuals who are infected with HIV should start ART immediately, making all populations and ages eligible for treatment. All HIV-exposed infants should also receive a regimen for ART meant for prophylaxis. As these guidelines are implemented and access to ART for children scales up worldwide, many more infants, children, and adolescents will be taking antiretroviral medications.

Adherence to ART is crucial to HIV management. Proper adherence to ART leads to lower viral loads, decreased symptoms in patients, and decreased viral resistance [4]. Viral resistance to first-line ART requires patients to switch to more expensive and less available second- and third-line therapies. As ART becomes more potent, it improves the immunological response for HIV-infected children, thus extending their life expectancy. With good ART adherence, HIV-infected children can live long, healthy lives; therefore, it becomes a priority to study and address the causes of poor adherence with the goal of maintaining successful therapy for as long as possible during a child's lifelong treatment.

Many barriers prevent children with HIV from maintaining good adherence. These include cost of medications, access to medications, stigma associated with HIV, and disclosure of HIV to children [5, 6]. However, an underlying theme within the barriers to ART adherence studies is the lack of pediatric-friendly formulations. Current pediatric ARTs are often unpalatable to children of all ages [7]. All these factors influence adherence and, therefore, survival of children with HIV.

Developing child-friendly formulations of ART will make it easier for caregivers to administer medications to children and easier for children to take medications. Both strategies will increase adherence, which is critical to successful long-term HIV therapy. This paper will review the current obstacles preventing adherence to pediatric ART, the barriers to creating more acceptable pediatric formulations, and evidence supporting the need for appropriate pediatric formulations of ART.

## 2. Four Main Obstacles Preventing Adherence to Current Pediatric Antiretroviral Therapy

### 2.1. Palatability

Poor palatability is directly associated with poor adherence in children with HIV [8–10]. Studies report that caregivers have difficulty administering medication to children due to the bitter taste of the medications [11, 12]. Poor palatability creates a struggle between children and their caregivers, frequently causing caregivers to take on the role of persuasive diplomats during medication administration [13]. This struggle adds to the burden that caregivers experience when providing care to their HIV-infected children. One study found that 81% of caregivers identified better tasting medications as the most important innovation needed to increase adherence [14].

The World Health Organization recommends lopinavir/ritonavir (LVP/r) as the first-line ART to initiate in children three years of age and younger and recommended it as the second-line treatment for children three years of age and older [15]. Protease inhibitors (PIs), such as lopinavir and ritonavir, are clinically effective in young children, and many are available in liquid forms, which eases dosing in children who are unable to swallow tablets [16]. However, poor palatability prevents PIs from having optimal benefits in HIV-infected children. Many studies describe the bitter taste associated with protease inhibitors such as LVP/r [17–19]. This bitter taste has been found to impact adherence in young children, leading many authors to call for new and innovative ways to administer PIs [20–22]. Developing more child-friendly formulations of protease inhibitors, particularly formulations that taste reasonably well, is a crucial need for children living with HIV.

### 2.2. Regimen Complexity

In addition to poor palatability, large pill burden has also been found to decrease adherence to ART [23, 24]. Many children have to take three or more pills or a combination of pills and liquids, every day, twice a day. The advent of fixed-dose combinations (FDCs), which combine two or three antiretroviral medications into one pill, has been shown to increase adherence in older children who are able to swallow pills [25, 26]. Few FDCs exist in nonpill form, therefore limiting their administration to older children who are able to swallow large pills [27].

In addition to FDCs, other ways of simplifying dosing regimens, such as once-a-day formulations, have also been shown to positively affect medication compliance. A study of extended-release, nevirapine-based ART, which can be given once a day, showed good immunologic response and improved adherence in children [28]. These formulations significantly increase dosing intervals, while simultaneously decreasing the hassle of medication administration for caregivers. The once-a-day formulations have been found to be preferable to FDCs and liquids [24]. It is therefore important to strive for FDCs that can be safely administrated once a day to children as a way to simplify ART administration and decrease pill burden.

Liquid formulations of ART were originally created for infants and children with HIV to ease administration by caregivers. Young children are not generally physically capable of safely swallowing pills or tablets. Nonetheless, liquid formulations have many shortcomings of their own. In addition to the poor palatability of liquid ARTs, they often require caregivers to measure out and administer precise amounts of the liquid. This can be a challenge: one study found that 80% of caregivers preferred tablets over liquids due to the inability to measure accurate doses, easy spillage during administration, and the large quantities they needed to administer [9]. Many liquid formulations also require refrigeration, which can become a huge problem in resource-limited settings where electricity is not always available in patients' homes. In the current WHO requirements, for children under three years of age, three separate liquid medications must be measured and administered daily based on weight, presenting no small challenge for their caregivers [15].

### 2.3. Swallowing

Because of the aforementioned complexity of liquids, caregivers often switch to pills as early as possible. However, many studies have found swallowing pills to be a barrier to adherence in children [10, 29, 30]. Studies have recorded how caregivers take measures into their own hands to overcome these challenges, documenting that they open capsules, crush tablets, and sprinkle contents into food [31, 32]. Crushing pills or opening capsules can reduce the bioavailability of the ART because the entire contents may not be administered, thus significantly reducing the targeted therapeutic exposure [30]. This may reduce viral suppression and promote viral resistance.

Many studies have looked into alternatives to swallowing large pills for children with HIV. Potential innovations to decrease the struggle between caregivers and their children include the following: recommendations to open capsules, combining medications with food, and knowing which pills are dispersible in water [33].

In another approach to this challenge, pill-swallowing training for HIV-infected children has been shown to increase ART adherence [19]. Gastrostomy tube insertion for medication delivery is a more drastic and invasive solution to administering ART to children who are unable to swallow pills or liquids [34]. These examples all support the need for easy-to-swallow medication options, such as sprinkles or more palatable flavored liquids for young children, to increase adherence to ART.

### 2.4. Storage and Transportation

A significant barrier to ART compliance is the requirement for complex storage and transportation. Many of the liquid protease inhibitors require cold-chain storage. This requires both the clinic and the patient to have a refrigerator to store the medications. In resource-limited settings, such as sub-Saharan Africa, where pediatric HIV is most prevalent, this might be an impossible requirement [35]. The World Bank estimates that only 23% of Kenyans, 18.2% of Ugandans, and 15.3% of Tanzanians have access to electricity in their homes [36]. Often, caregivers opt for tablet medications which they will crush or they improperly store liquid formulations, both of which lower bioavailability of the medications. A study reported that 63% of caregivers favored innovations of medications that did not require refrigeration [14].

In addition, caregivers often must transport large volumes of liquid ART for their children to have a monthly supply or more. This can be significant barrier for caregivers living in rural areas who may walk many kilometers to arrive at the clinic. For example, a 10 kg toddler on first-line ART (ABC + 3TC + LVP/r) would take 28 mL of ART daily, which is equal to about two and a half liters every three months. This requires caregivers to carry seventeen 160 mL bottles home from the clinic every three months. Due to the stigma associated with HIV, caregivers aim to hide medications from their community, which is difficult when caregivers must carry so many bottles. A study in Uganda found that 63% of caregivers complained about the weight of bottles as being a major barrier [37]. Large quantities of bottles or large bottles are difficult to conceal and carry when traveling home from the clinic, providing yet another reason why many caregivers prefer to switch to tablets for their children as early as possible [38]. It is therefore important to design HIV medication formulations that are easy to transport and store.

## 3. Barriers to New Pediatric Formulations

### 3.1. Reducing Vertical Transmission Will Reduce New Cases of Pediatric HIV

The world aims to eradicate mother-to-child transmission of HIV, therefore eliminating child cases of HIV and subsequently the need for pediatric formulations. This may be seen to significantly reduce the impetus to develop new pediatric ART formulations. Although the elimination of pediatric HIV is a shared goal among healthcare providers, pharmaceutical companies, and NGOs around the world, it will take many years before vertical transmission is prevented. For now, millions of children are still living with this disease. Until this objective is met, children need access to child-friendly formulations of ART [39]. Moreover, a considerable number of children are still unable to access these medications [40].

Many factors continue to drive the need for pediatric ART, including the challenges of successfully implementing PMTCT and scaling up widespread access to pediatric ART. The risk of perinatal transmission remains at 2%, even if a woman is on ART, has a low or undetectable viral load, follows the recommended treatment regimen, and does not breastfeed [41, 42]. Resource-limited settings, where there is limited access to healthcare and baby formulas, may not be able to even reduce their rates of perinatal transmission to as low as 2% if they are not able to address other challenges in healthcare delivery systems. These challenges include a severe lack of healthcare workers, poor implementation of appropriate HIV care and prevention guidelines, and insufficient funds budgeted nationally for healthcare [43, 44]. In addition, the WHO recommends breastfeeding for all mothers, regardless of HIV status, with mothers on ART throughout the breastfeeding period and HIV-exposed infants on some ART for a portion of this time [41]. Thus, both prevention efforts for HIV-exposed infants and treatment for HIV-infected children continue to require pediatric formulations to aid in the eradication of pediatric HIV.

### 3.2. Inadequacies in Supply Chain and Healthcare Delivery Systems

Surveys of the pediatric ART market review that outdated procurement practices and gaps in supply chains are often responsible for low uptake of innovative or new pediatric drug formulations [40, 45, 46]. While some of these gaps in access to the formulations are attributed to high costs for new medications, other relevant barriers include antiquated procurement practices and stagnant government policies in the face of changing guidelines for treatment [47, 48]. The introduction of effective pediatric ART innovations will not make any impact on children's health unless these supply chain challenges are addressed and steps are made to assess national barriers to uptake for particular formulations, the level of education or acceptance around new ART regimens, and country-level policies and practices that will impact access and implementation. Incorporation of innovative pediatric formulations into international and national treatment guidelines and policies, such as the Essential Medicines WHO Model List, may assist in the scale-up and stability of these treatment regimens [48]. At a national level, Ministries of Health may need to work towards shaping forward-looking, sustainable supply chain infrastructures that can adapt to both changes in available therapy and in healthcare system delivery [48].

### 3.3. Poor Incentives for Pediatric Formulation Development

In its “Developing an Optimized List of Pediatric ARV Formulations” report, the WHO presents the argument that since children only make up 7% of HIV cases worldwide, increases in the number of formulation options will decrease the demand for currently available formulations. This could lead to market instability and therefore a lack of incentive for investment in the pediatric ART market [46]. On top of this challenge is the previously discussed (and critically important) global push to decrease the number of child HIV infections and therefore to decrease the numbers of children requiring ART, which also takes away pharmaceutical companies' incentive to pursue new options for children. Nonetheless, more than 3 million children will need to continue to take ART even once the AIDS-free generation goals are met.

Incentives are required to drive further investment in research and development for pediatric formulations and innovations that have been shown to increase adherence. Recognizing the potential lack of incentives for this work, recent initiatives such as Drugs for Neglected Diseases Initiative (DNDi), the NIH's Development of Appropriate Pediatric Formulations and Pediatric Drug Delivery Systems RO1, and the Accelerating Children's HIV/AIDS Treatment (ACT) initiative supported by the Presidents Emergency Plan for AIDS Relief (PEPFAR) and the Children's Investment Fund Foundation (CIFF) have created incentives for the investment in pediatric ART formulation development [49–51]. These efforts must be sustained and similar incentives created to fund research and development of needed pediatric medications.

### 3.4. Pharmacokinetics and Development Challenges When Designing Innovative Formulations

Pharmacokinetics, toxicity, and delivery preferences for pediatric medications can differ greatly from adult medications, requiring specific research to evaluate the safety and efficacy of pediatric medications [52, 53]. This research often goes undone since developing child-friendly medications is a challenging and lengthy process that requires ensuring quality, safety, and efficacy. Pharmacokinetic properties can vary according to age and weight, which requires new drug formulations to have flexible dosages [52, 54]. Palatability is a very important factor when developing innovative medications, but additives can cause toxicity or change the pharmacokinetics of certain medications [55–57]. Developing critical innovations for certain populations, such as liquid formulations of FDCs, can be limited for certain compounds that are large or insoluble molecules or can run into dosing challenges when the compounds do not have similar scaling as the child moves across weight bands. Combinations may also result in further pharmacokinetics challenges produced by their own interactions. The extensive research required to ensure the safety of new medications can deter researchers and investigators from studying new and innovative pediatric antiretroviral medications; however, many of the innovations currently being studied show promising results [58–61].

### 3.5. Psychosocial Factors That Influence Adherence

In addition to lack of pediatric formulations, there are many other factors that influence adherence to ART. Medication properties that influence adherence include poor palatability, large pill size, regimen complexity, and short dosing intervals, but these properties are only encountered if the child is given the opportunity to take the medication. Stigma, lack of medical literacy, disclosure status of the child, child-caregiver relationship, and high costs of HIV medications also prevent children from receiving treatment or being compliant [5]. Even after developing new pediatric formulations, these causes of poor adherence will still remain. Despite these additional barriers, it is important to increase the number of child-friendly antiretroviral drugs. Increasing the available options for ART will diminish one of the compounding factors for poor adherence.

## 4. Benefits of Child-Friendly ART

### 4.1. Increased Adherence

The link between adherence and formulation properties is the most poignant argument for the development of child-friendly ART. Many studies show a significant connection between adherence and ART attributes such as palatability, pill size, pill burden, short dosing intervals, and regimen complexity. Over 21% of caregivers in a South African study reported difficulties giving medication due to poor palatability [12]. In Italy, a similar study found that 38% of caregivers reported trouble administering ART due to palatability and pill size [62]. A study of 119 children with HIV in Canada demonstrated that 1/3 of participants failed to adhere to the medication regimen due to palatability [63]. Numerous studies, such as these, report that current ART formulations worsen adherence [64–67].

The significant impact that HIV medication formulations have on adherence has led to the study of alternatives and new formulations to help with medication administration in children. Studies have looked at how to decrease poor palatability by adding flavoring and creating pH-sensitive microparticles to hide the bitter taste of many ARTs. A study in Thailand examined the addition of a flavoring agent to generic ART, using FLAVORx®, whose ingredients include a flavoring agent, propylene glycol, ethyl alcohol, water, and triacetin, in ten different flavors: strawberry, orange, banana, grapes, bubble gum, watermelon, lemon, cherry, vanilla, and chocolate [31]. They found that 80% of caregivers reported easier medication dosing to children with the flavoring addition, and the most popular flavors in this study were strawberry, orange, and grape [31]. Another study loaded indinavir into pH-sensitive microparticles, preventing children from experiencing the bitter taste of the protease inhibitor [59]. Researchers in another study mixed flavoring into liquid EFV, with an aim to decrease the burning mouth syndrome suffered by many children who use this medication in liquid form. The study found that these new flavored formulations decreased burning mouth syndrome in healthy adult volunteers [60]. A separate study evaluated the effectiveness of a milk-based powder formulation of ritonavir to ease administration to infants and children. It found that casein micelles, a milk protein, are an efficient carrier system of ritonavir and serve to decrease bitter taste [68].

In addition to manipulating taste, other innovators have been studying the impact of different modes of administration. These modes include sachets that deliver liquid or gel ART all at once, sprinkles and nanoparticles that allow caregivers to mix medications with food or liquids, and dissolvable tablets that can be incorporated into fluids. Nanotechnologies have been used to administer insoluble LPV/r in sprinkles or sachet formulations and have shown stability and good bioavailability [58]. Two articles studied the effectiveness of novel sprinkle formulations of LPV/r and efavirenz (EFV) [66, 69]. One study of sprinkle use in Uganda found that over 70% of the participants in two of three cohorts chose to continue sprinkle formulations over liquid formulations after an 8-week trial [69]. Sprinkles have been found to improve tolerability of first-line ART in children and reduce the cost to treat HIV-positive children [16]. Scored dispersible lamivudine/stavudine combination tabs have been shown to be cost-effective and easier to administer by caregivers [70]. In addition, Drugs for Neglected Diseases Initiative and Cipla joined to devise a sprinkle and 4-in-1 sachet to ease delivery of ART in children [49]. The recent development of ART-delivering sachets developed by Pratt School of Engineering at Duke University has reduced the number of perinatal HIV infections among infants in Ecuador [71]. Similar innovations can be used to deliver ART to infants with HIV.

Because fixed-dose combinations and once-a-day formulations are associated with better adherence, innovators have also aimed to create innovative solutions for children to help decrease pill burden. LPV/r, which has been recommended as first-line treatment by the WHO, has been combined with abacavir/lamivudine or zidovudine/lamivudine in a study to create a more tolerable, first-line, fixed-dose combination for children [72]. Another study assessed the ability to combine ritonavir and darunavir as a dry powder as a second-line treatment for children [73]. A study aiming to find a FDC effective in infants younger than 6 months devised a FDC suppository with stavudine, lamivudine, and nevirapine, which can ease administration in fussy infants [74]. Nonetheless, few nonpill options are available in fixed-dose combinations for infants and young children [27], likely due to the previously discussed challenges with cost of development and pharmacokinetics and dosing challenges.

In addition to FDC and once-a-day formulations, long-acting formulations of ART, such as once-a-month injectable antiretroviral and weekly ART infusions, are on the horizon and could help children and adults who struggle with adherence to daily therapy [75–77]. Though injections can decrease pill burden for children, cold-chain storage requirements for injectable medications might pose similar barriers to those for liquid medications in resource-limited settings. On the other hand, the potential for not only improving ART adherence but also exploring attempts to synchronize injectable antiretrovirals with long-acting injections for contraception could be particularly interesting for adolescent females.

Increased adherence in children with HIV has benefits for both the child and the larger HIV-positive community. Proper adherence leads to sufficient viral suppression, which leads to longer and healthier lives for children living with HIV. In addition, proper adherence reduces viral resistance [78]. Viral resistance can be devastating in the community, requiring individuals to change to a different ART, exhausting the available medications. It is therefore imperative to introduce new and innovative therapeutic options for children to increase adherence and decrease viral resistance.

### 4.2. Easier Dosing for Caregivers

In addition to better adherence in children, pediatric formulations of ART have the possibility of reducing the time and energy investment required of caregivers to administer medication to their children. Many studies have reported that a significant cause of nonadherence is child refusal [18, 79]. The struggle between child and caregiver can be taxing for elderly caregivers and single mother and fathers. Innovations such as taste-masking, sprinkles, or injectable, long-acting agents could help ease the struggle between caregivers and the children for whom they care.

Concealing and hiding medications on the go is important for both caregivers and children as a way to reduce stigma and bullying. Sachets, such as the Pratt Pack created by Duke University, can ease transportation of medications and aid caregivers in concealing medications [71]. As discussed previously, liquid medications can be easily spilled and time-consuming for caregivers to measure out for dosing; innovations such as sprinkles, dissolvable tablets, and sachets can reduce the mess and time it takes to prepare medications. Therefore, the development of child-friendly formulations would be beneficial for children and their caregivers.

## 5. Future of Pediatric Antiretroviral Therapy

Despite many innovations, pediatric formulations are still very limited in comparison to the number of adult formulations [80, 81]. Without adequate or convenient pediatric formulations, prescribers and caregivers resort to cutting, crushing, or substituting adult medications to meet the need for easier administration of medications for children [26, 32, 38, 61]. This can have drastic, negative impacts on children, leading to over- or underdosing [82].

The pediatric market for ART is not a small version of the adult market, but rather a unique niche which requires developers to consider different routes and methods of administration, different strengths, and a variety of flavors [40, 83]. Fixed-dose combinations and once-a-day medications have solved many issues of adherence in older children and adolescents who are able to swallow pills. For infants and young children, there is still a lack of accessible formulations. This results in poor adherence, viral resistance, and decreased survival of HIV-positive children.

Many organizations, including UNITAID, Drugs for Neglected Diseases initiative (DNDi), Medicines Patent Pool (MPP), Clinton Health Access Initiative, President's Emergency Plan for AIDS Relief, and Elizabeth Glaser Pediatric AIDS Foundation, have called for the prioritization of pharmaceutical companies to address the need for new innovative formulations [84–87]. As the WHO aims to eliminate pediatric transmission of HIV, it is important not to neglect the 3.2 million children with HIV worldwide and the countless children who require ART treatment during breastfeeding to achieve WHO goals and prevent new child infections.

To improve the lack of pediatric formulations, the World Health Organization launched the global campaign “Make Medicines Child Size.” The case is even more critical in the case of poverty-related diseases that mainly affect children in poor countries, such as HIV [88]. Due to the large number of children with HIV worldwide, the chronicity of the disease, and the critical role of adherence to therapy, it is imperative to provide child-friendly medications to improve adherence and therefore increase life expectancy and success of treatment in HIV-positive children.

## 6. Conclusion

Over 3.2 million children worldwide are waiting for pediatric formulations of antiretroviral drugs. To address this need, innovations in pediatric formulations must strive towards (1) safe and effective ART for children, (2) palatable and easy-to-swallow medications, (3) fixed-dose combinations to decrease pill burden, (4) once-a-day formulations to lengthen dosing intervals, (5) medications that are easy to transport and store, (6) formulations that are simple for caregivers to administer, and (6) securing incentives to drive investments in the development of these critical formulations. Investment must be made in pediatric-friendly formulations, especially for infants, toddlers, and young children, to allow this generation to survive and thrive.
